# Maternal temperature exposure impairs emotional and cognitive responses and triggers dysregulation of neurodevelopment genes in fish

**DOI:** 10.7717/peerj.6338

**Published:** 2019-01-31

**Authors:** Violaine Colson, Morgane Cousture, Danielle Damasceno, Claudiane Valotaire, Thaovi Nguyen, Aurélie Le Cam, Julien Bobe

**Affiliations:** Fish Physiology and Genomics, INRA LPGP UR1037, Rennes, France

**Keywords:** Behavior, Egg, Transcriptome, Trout, Stress, Temperature, Intergenerational, Cognition, Emotion, *Auts2*

## Abstract

Fish are sensitive to temperature, but the intergenerational consequences of maternal exposure to high temperature on offspring behavioural plasticity and underlying mechanisms are unknown. Here we show that a thermal maternal stress induces impaired emotional and cognitive responses in offspring rainbow trout (*Oncorhynchus mykiss*). Thermal stress in mothers triggered the inhibition of locomotor fear-related responses upon exposure to a novel environment and decreased spatial learning abilities in progeny. Impaired behavioural phenotypes were associated with the dysregulation of several genes known to play major roles in neurodevelopment, including *auts2* (autism susceptibility candidate 2), a key gene for neurodevelopment, more specifically neuronal migration and neurite extension, and critical for the acquisition of neurocognitive function. In addition, our analysis revealed the dysregulation of another neurodevelopment gene (*dpysl5*) as well as genes associated with human cognitive disorders (*arv1*, *plp2*). We observed major differences in maternal mRNA abundance in the eggs following maternal exposure to high temperature indicating that some of the observed intergenerational effects are mediated by maternally-inherited mRNAs accumulated in the egg. Together, our observations shed new light on the intergenerational determinism of fish behaviour and associated underlying mechanisms. They also stress the importance of maternal history on fish behavioural plasticity.

## Introduction

In the current context of global climate warming, wild and aquaculture fish are at increasing risk of being exposed to varying environmental factors including suboptimal temperatures at specific periods of their lifecycle. Fish are highly sensitive to extreme or abnormal (i.e., outside of the normal physiological range) temperatures throughout their lifecycle, even for short periods of time. This is especially true for key periods such as the reproductive period, during which the female gamete undergoes final oocyte maturation. The direct impact on gamete quality has been thoroughly investigated in many temperate species (see Bobe & Labbe, 2010; Kjorsvik, Mangorjensen & Holmefjord, 1990; Migaud et al., 2013 for review). Exposure of mature female fish to high temperature during reproductive season (i.e., prior or around the time of ovulation) has a dramatic impact on egg size (Jonsson & Jonsson, 2016), egg viability and subsequent embryonic success (Aegerter & Jalabert, 2004) including reduced survival throughout development. Despite this well documented negative impact on egg quality and subsequent embryonic development, the long-term effects of maternal exposure to suboptimal temperature on progeny behaviour and phenotypic plasticity remain unknown. More specifically, the intergenerational consequences of mother exposure to abnormal temperature on offspring emotional responses and cognitive performances—two key components of animal welfare (Boissy & Erhard, 2014; Boissy, Veissier & Roussel, 2001; Désiré, Boissy & Veissier, 2002; Duncan & Petherick, 1989; Duncan & Petherick, 1991)—have never been investigated.

Several studies have, however, shown that maternal history can impact offspring behaviour and phenotypic plasticity (i.e., ability of an organism to change its morphology, physiology, or behaviour according to stressful environmental conditions (Bijlsma & Loeschcke, 2005). This intergenerational effect on offspring behaviour was observed in salmonid fish in which stress during reproductive season, or at least artificial exposure to stress hormones, has a significant intergenerational impact on offspring phenotypic plasticity, including modifications of cognitive abilities (Sloman, 2010) and emotional reactivity (Colson et al., 2015b; Eriksen et al., 2011; Espmark et al., 2008). In contrast, the underlying mechanisms mediating these effects remain poorly documented. In mammals, profound long lasting behavioural deficits have been observed in mice originating from stressed mothers, possibly due to epigenetic modifications occurring in the mother and transmitted to offspring (Weiss et al., 2011). In fish, a recent study has demonstrated the existence of the programming of stress axis function in zebrafish (*Danio rerio*) offspring by maternal social status (Jeffrey & Gilmour, 2016). Another study showed that three-spined stickleback (*Gasterosteus aculeatus*) embryos respond to maternal exposure to predation risk via changes in gene expression (Mommer & Bell, 2014). A better understanding of underlying mechanisms is, however, necessary to fill the gap between maternal history and offspring phenotypic plasticity. Identifying molecular players mediating intergenerational effects will contribute to identifying the mechanisms underlying the neural changes leading to a change in fear response and learning capacity.

The aim of this study was to thoroughly characterize the impact of high temperature exposure of female rainbow trout (*Oncorhynchus mykiss*) during the reproductive season on offspring emotional and cognitive phenotypes, using specific behavioural tests previously validated in the laboratory (Colson et al., 2015a; Poisson et al., 2017; Sadoul et al., 2016). We also aimed at deciphering the molecular mechanisms mediating such intergenerational effects by analysing genome-wide gene expression in eggs and developing embryos following maternal exposure to high temperature.

## Material and Methods

### Ethics statement

Fish were reared in INRA LPGP facilities, which hold full approval for animal experimentation (C35-238-6). All fish were reared and handled in strict accordance with French and European policies and guidelines of the INRA LPGP Institutional Animal Care and Use Committee, which specifically approved this study (no. T-2016-55-VC-CV).

### Maternal treatment and fertilization

**Figure 1:**
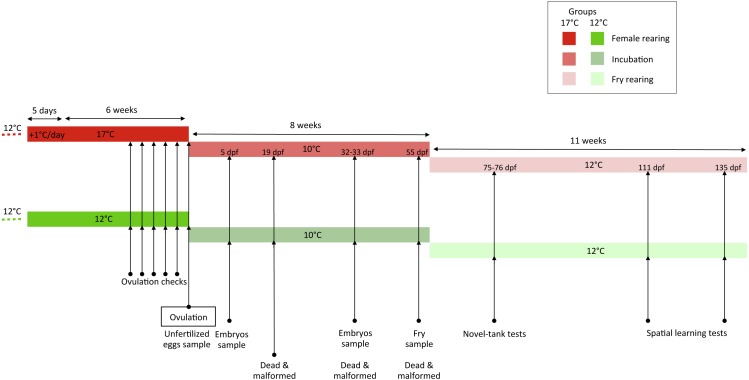
Experimental schedule.

Two-year old female rainbow trout were exposed to either 12 °C (12 °C group, standard reproduction conditions) or 17 °C (17 ° C group, high suboptimal temperature) for six weeks before ovulation (Experimental schedule is given in Fig. 1). The temperature of 17 °C was selected because it is known to induce a dramatic decrease in embryonic survival (Aegerter & Jalabert, 2004) and to correspond to actual aquaculture situations, especially in photoperiod-induced out of season spawning during summer. For each group, 30 marked (external tag placed on the dorsal fin) females were kept in 2.5 m^3^ tanks (2 × 2 × 0.62 m, length × width × water height). In the 17 °C group, females initially reared at 12 °C were acclimated for five days to an increase of 1 °C/day until 17 °C. For three weeks before ovulation, females were checked every two-three days to detect ovulation, by applying gentle pressure to the abdomen under anaesthesia (females transferred to a 100-l tank containing water circuit + 100 ml tricaïne + 100 ml bicarbonate of sodium). In both experimental group, eggs originating from four simultaneously ovulating females of each group were collected and fertilized using a pool of sperm collected from four males held at 12 °C. The use of males held under optimal conditions, including normal temperature is standardly used in gamete biology studies in fish and other vertebrates (Cabrita, Robles & Herráez, 2009). Fertilization was performed immediately in both groups in order to avoid any bias on subsequent behavioural phenotypes that would have been induced by differences during embryo development. For each female, fertilization of 800 eggs was performed at 10 °C using sperm extender ActiFish medium (IMV, L’Aigle, France; 100 ml ActiFish + 400 ml water) and fertilized eggs were distributed within a tray (20 × 50 cm) in two incubators (10 ×10 cm) (approximately 400 eggs/incubator and two incubators/tray) supplied with 10 °C flow-though recycled water. Each tray was covered with a lid to avoid exposure to light.

### Monitoring of developmental success

Developmental success was monitored at eyeing stage, i.e., 19 days post-fertilization (dpf), hatching (32–33 dpf), and completion of yolk-sac resorption (YSR, 55 dpf) by counting dead embryos that were removed from incubators (Fig. 1). The occurrence of malformations was obtained by taking a picture of euthanized malformed fry in each incubator at YSR. The types of malformed fry observed in this study were: torsion (T), yolk sac resorption defects (YSD) and other malformations (O) as described in (Bonnet, Fostier & Bobe, 2007a). For each female, the occurrence of each type of malformations was calculated in comparison to the total number of malformed fry. Percentages of mortalities and malformations per incubator were obtained by counting the final number of live fry at swim-up stage, before transfer into rearing tanks.

### Sample collection during embryo development

In both experimental groups, and in all egg clutches, biological samples were collected at four different stages (Fig. 1): unfertilized eggs (0 dpf), following zygotic genome activation (Nagler, 2000) (5 dpf), hatching after removing yolk-sac (32–33 dpf), and YSR (55 dpf), which also corresponded to the stage of behavioural phenotyping. Entire (i.e., whole body) embryos were sampled. All samples were frozen in liquid nitrogen and held at −80 °C until further processing.

### Fry rearing

After yolk-sac resorption, at 55 dpf (Fig. 1), swim-up fry originating from the two incubators of each female were combined and transferred to seven distinct tanks (50 × 60 × 28 cm) (approximately 200 fry/84 L), corresponding to seven different females (four from the 12 °C group and three from the 17 °C group). The mortality rate of one of the 17 °C female was 98.4% and we did not obtain enough offspring to perform behavioural phenotyping. For this female, we however sampled remaining fry to perform transcriptome analyses. Water temperature was maintained at 12 °C. Fish were fed manually four times a day on a commercial diet (Biomar, 48% protein and 22% lipid, 0.5 mm diameter pellets). Tanks were automatically illuminated from 8:00 to 20:00. Before each behavioural test, fish were starved for 24 h. At the end of each test, fish were netted and transferred into individual bowls containing 250 ml of the tank water to which a lethal dose of anaesthetic (tricaïne: 4.5 ml + bicarbonate of sodium: 5 ml) had been added.

### Phenotyping of offspring behaviour

For each female (three 17 °C females and four 12 °C females), different fry were subjected to the following behavioural tests thoroughly described in Poisson et al. (2017).

### Assessment of offspring emotional reactivity

Fish propensity to express fear-related behaviour (e.g., emotional reactivity) was evaluated individually in a novel-tank test (social isolation in a novel tank) at 75–76 dpf (Fig. 1). The novel tank (30 × 19 × 16 cm) was supplied with 12 °C flow-though recycled water. Fifteen fish per female, randomly netted either at the top or the bottom of the water column, were observed. The treatment order was randomly chosen.

Behavioural responses were video-recorded for 30 min. We analysed the first (0–5 min) and the last (25–30 min) 5-min intervals with EthovisionXT software (Noldus, Netherland). The following behavioural parameters were calculated for each individual: total distance travelled (cm), maximum swimming velocity (cm/s), angular velocity (° /s) (i.e., erratic swim), and time spent (%) in the border zone (i.e., thigmotaxis) corresponding to the mean length of all fish tested (3.51 ± 0.03 cm). At the end of the test, the body weight (W) and length (L) were measured. For each fish, the condition-factor was calculated as followed: K-factor = 100 (W/L^3^3).

### Assessment of offspring spatial learning abilities

Between 111 and 135 dpf (Fig. 1), offspring propensity to locate a food-rewarded arm was assessed in a T-maze supplied with 12 °C flow-though recycled water (see Poisson et al., 2017) for a complete setup description). Five randomly netted fish per female were tested. After 24 h of food deprivation and 30 min of acclimation in the start-box of the T-maze, a remote-controlled guillotine door was pulled-up and fish position in the T-maze was video-recorded. A visual cue (black cross) was located on the wall of the T-maze at the entrance of the reward arm. When the fish crossed an invisible line separating the rewarded arm from the rest of the maze, a mechanic ridge, remotely-controlled by an experimenter observing live videos in an adjacent control room, released pellets. Then the fish was left to eat the pellets for at least 5 min before being gently netted and introduced in its individual holding tank until the next trial. Eleven successive trials were run for four consecutive days (two trials on the first day and three on the other days). The treatment order was randomly chosen on the first day. We measured the latency to leave the start-box (Latency SB), the latency to reach the reward arm after the fish had left the start-box (e.g., right choice, Latency RC) and the ability of the fish to make the right choice (i.e., to choose the rewarded arm first after leaving the start-box). We counted the number of fish making the right choice first in less than 900 s in at least four out of the last seven trials (57% correct choices).

### Gene expression profiling

Transcriptome analysis was conducted using four egg batches originating from females held at 12 °C and four egg batches originating from females held at 17 °C, with the exception of YSR/12 °C for which only three RNA samples of sufficient quality could be obtained. RNA was extracted from 20 eggs sampled at fertilization, 20 eggs at 5 dpf, six embryos at hatching and six fry sampled at YSR. Each sample was collected randomly (either at the border or the center of the incubator) or randomly when hatched fish were swimming. Frozen tissues were lysed with a Precellys Evolution Homogenizer (Ozyme, Bertin Technologies) in TRI Reagent (TR118; Euromedex) and total RNA was extracted according to the manufacturer procedure and followed by Nucleospin RNA isolation kit (740955; Macherey Nagel). Gene expression profiling was conducted using an Agilent 8 × 60 K microarray (GPL24910) as previously described (Zarski et al., 2017). Samples were randomly distributed on the microarray for hybridization. The data were processed with GeneSpring software (Agilent v.14.5) using gMedianSignal values. After data processing, one sample from the hatching/17 °C group, which behaved differently from other samples, even after normalization, was removed from subsequent analysis. Corresponding data were deposited in Gene Expression Omnibus (GEO) database under the reference GSE113377.

### Statistics

Due to low number of simultaneously ovulating females, percentage mortalities and malformations were compared between treatments using nonparametric Mann–Whitney tests (R, Mann–Whitney-Wilcoxon non-paired tests).

Fish length was analysed after taking into account the temperature as a fixed factor (two levels: 12 °C and 17 °C) and the females as a random factor. A generalized linear mixed model (GLMM) was fitted using the nlme package in R 3.3.1 (R Core Team, 2016), and by assuming a normal distribution. Significance of the random effect was checked using the 95% confidence interval of the variance, 0 being excluded of the interval in case of significance.

The analyses of the novel tank test consisted in testing the effect of the temperature, the effect of the interval (two levels: 0-5 min and 25–30 min) and their interaction on each dependant variable. The mothers and individuals (repeated measures) tested within treatments and intervals were defined as random factors in our statistical model. Distance travelled and maximum velocity were square root transformed, while angular velocity was log-transformed in order to reach normality and to fix GLMMs models using the nlme package. Significance of the random effect was checked using the 95% confidence interval of the variance, 0 being excluded of the interval in case of significance. When models were significant, post-hoc analyses were performed using HSD-Tukey tests. For thigmotaxis, data were too far from a normal distribution so we fixed a GLMM using the lme4 package, assuming a gamma distribution with inverse function. With this R package, a low variance associated with the random factor female indicated non-significant random effects.

Correlations between all parameters were evaluated using a Pearson test in R software.

The analyses of the spatial learning test consisted in testing the effects of the temperature, the trial (considered as a covariable), and their interaction on each dependant variable. We fitted a GLMM model (using the nlme package) assuming first-order autocorrelation and specified “individual” nested under “female” as random effect factors to account for female effect and repeated observations of individual fish.

For all models, if there were non-significant effects on factors or interactions, stepwise backward eliminations were performed to sequentially simplify the full model. The models were validated using analysis of residuals (normality assessment). All the models and their respective results are presented in Table 1.

**Table 1:** Statistical models. Statistical models used for biometric data (variables: fish length and K-factor) and behavioural data obtained from the novel-tank test (variables: distance travelled, maximum velocity, angular velocity, and thigmotaxis) and the spatial learning test (variables: latency to leave the start-box, i.e., latency SB, and latency to make the right choice, i.e., latency RC). All analyses were performed with R software.

			Temperature			Interval			Temperature × Interval		
Variables	Package	R models	*d*.*f*.	*F*	*P*-value	*d*.*f*.	*F*	*P*-value	*d*.*f*.	*F*	*P*-value
Length	nlme	lme(length∼temperature, random =∼1—female)	1	4.15	*0.09*						
K-factor	nlme	lme(K.factor∼temperature,random =∼1—female)	1	2.63	0.16						
Distance travelled	nlme	lme(sqrt(distance)∼temperature*interval, random =∼1—female/individual)	1	0.002	0.96	1	6.78	*0.01*	1	5.26	*0.02*
Maximum velocity	nlme	lme(sqrt(velocitymax)∼temperature*interval, random =∼1—female/individual)	1	0.08	0.79	1	22.89	*<0.001*	1	0.33	0.56
		lme(sqrt(velocitymax)∼interval, random =∼1—female/individual)				1	23.06	*<0.001*			
Angular velocity	nlme	lme(log(angularvelocity)∼temperature*interval, random =∼1—female/individual)	1	0.34	0.58	1	0.79	0.37	1	7.60	*0.007*
**Variable**	**Package**	**R model**	**Estimates**	***t*-value**	***P*-value**	**Estimates**	***t*-value**	***P*-value**	**Estimates**	***t*-value**	***P*-value**
Thigmotaxis	lme4	glmer((thigmotaxis)∼temperature*interval + (1—female/individual), family =Gamma(link =inverse)) anova(model,test =”F”)	−0.19	−0.53	0.59	−0.27	−0.81	0.42	−0.09	−0.21	0.84
			**Temperature**			**Trial**			**Temperature**×***Trial***		
**Variables**	**Package**	**R models**	***d.f.***	**F**	***P*-value**	***d.f.***	***F***	***P*-value**	***d.f.***	***F***	***P*-value**
Latency SB	nlme	lme (latencySB ∼trial, random = ∼ + 1—female/individual, cor = corAR1())				1	62.27	*0.023*			
Latency RC	nlme	lme (latencyRC ∼temperature*trial, random = ∼+ 1—female/individual, cor = corAR1())	1	2.52	0.17	1	5.38	*0.02*	1	12.31	*<0.001*

We compared the proportions of fish making the right choice in less than 900 s in at least four out of the last seven trials by a chi-square test.

Differences were found to be significant when *P* < 0.05 and tendencies were considered for 0.05 <*P* < 0.1.

For microarray analysis, gene expression data was scale normalized and log(2) transformed before statistical analysis. The differences between the groups were analyzed using a two-way ANOVA with two factors (temperature, stage and their interaction), with a corrected *P*-value < 0.05 (Benjamini–Hochberg correction). For individual genes, non-parametric Mann–Whitney tests were performed between 12 °C and 17 °C groups within egg and 5 dpf stages to reveal any significant differential expression.

## Results

### Influence of maternal exposure to high temperature on developmental success and growth

**Figure 2:**
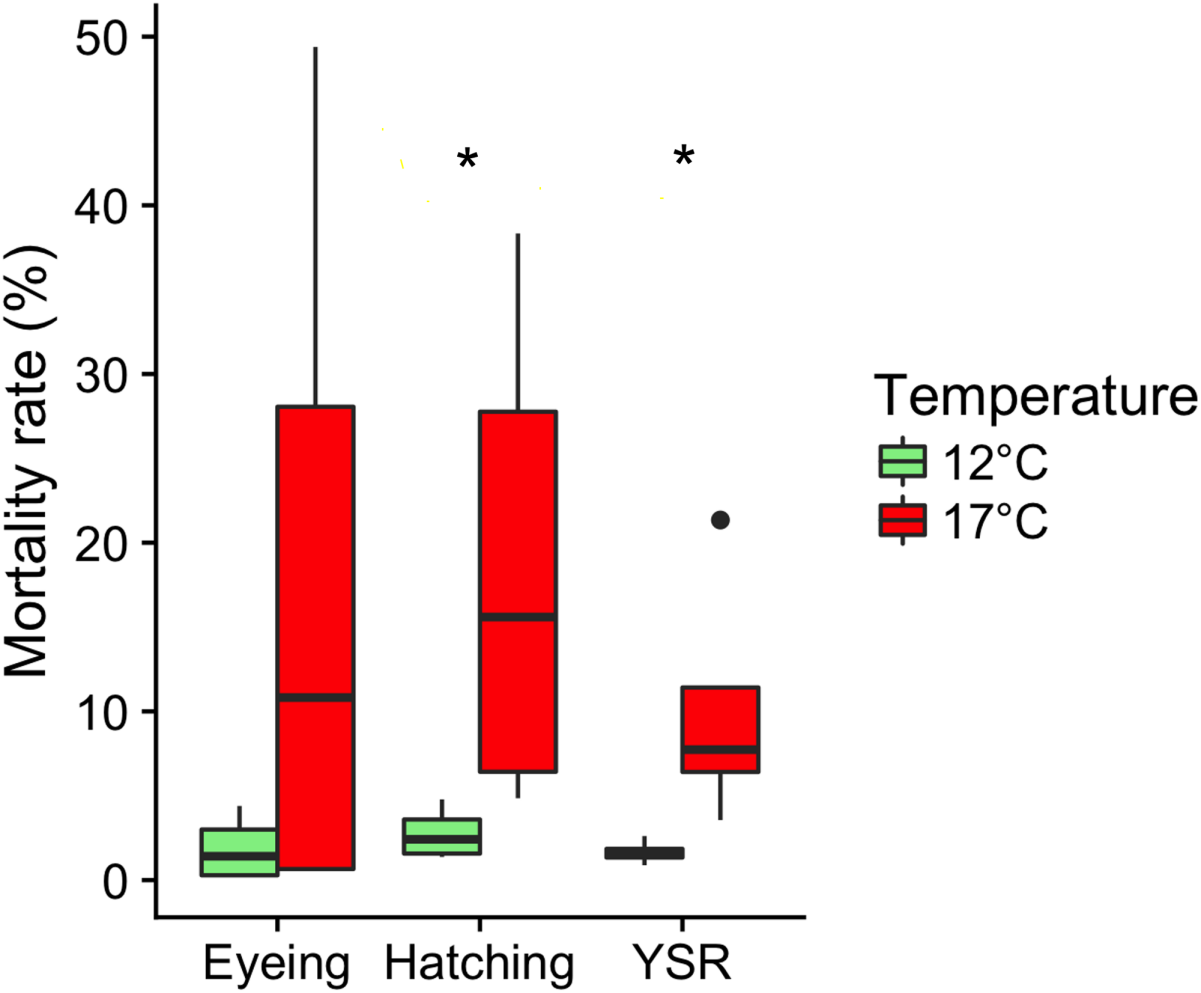
Embryonic mortalities. Effects of rearing temperature before ovulation (12 °C and 17 °C) on the occurrence of embryonic mortality (%) at different developmental stage (eyeing, hatching and yolk-sac resorption). Values are medians (quartiles: 25 and 75%) and data beyond the end of the whiskers are outliers and plotted as points. ^∗^*P* < 0.05: significant difference between treatments (*n* = 4).

Maternal exposure to high temperature had a major impact on offspring survival. A dramatic increase in mortality was observed throughout early development when eggs originated from females held at 17 °C even though this difference was not significant until hatching due to a high variability (Fig. 2). The overall median (quartiles: 25 and 75%) mortality rate was below 10% in the 12  °C group, while it was over 40% in the 17  °C group, with 6.62(5.30–7.59)% and 40.77(13.49–73.86)%, respectively (*W* = 0, *P* < 0.05). In contrast, no difference in the median (quartiles: 25 and 75%) occurrence of malformed fry was observed at yolk-sac resorption between the 12 °C and the 17  °C groups, with 7.57(6.44–8.76)% and 5.45(4.48–7.14)%, respectively (*W* = 11, *P* = 0.48). Similarly, the occurrence of the different types of malformation did not significantly vary among the experimental groups (Fig. S1). At 75 days post-fertilization, fish mean (± SEM) length tended to be lower in 17 °C than in 12 °C (3.44 ± 0.05 cm *vs.* 3.59 ± 0.02 cm), although not significantly (*F*_1,5_ = 4.15, *P* = 0.09). The condition-factor did not significantly differ between 12 °C and 17 °C fish (mean K-factor ± SEM: 0.99 ± 0.01 *vs* 0.95 ± 0.01; *F*_1,5_ = 2.63, *P* = 0.16). Model results are given in Table 1.

### Offspring behaviour in the novel-tank test

**Figure 3:**
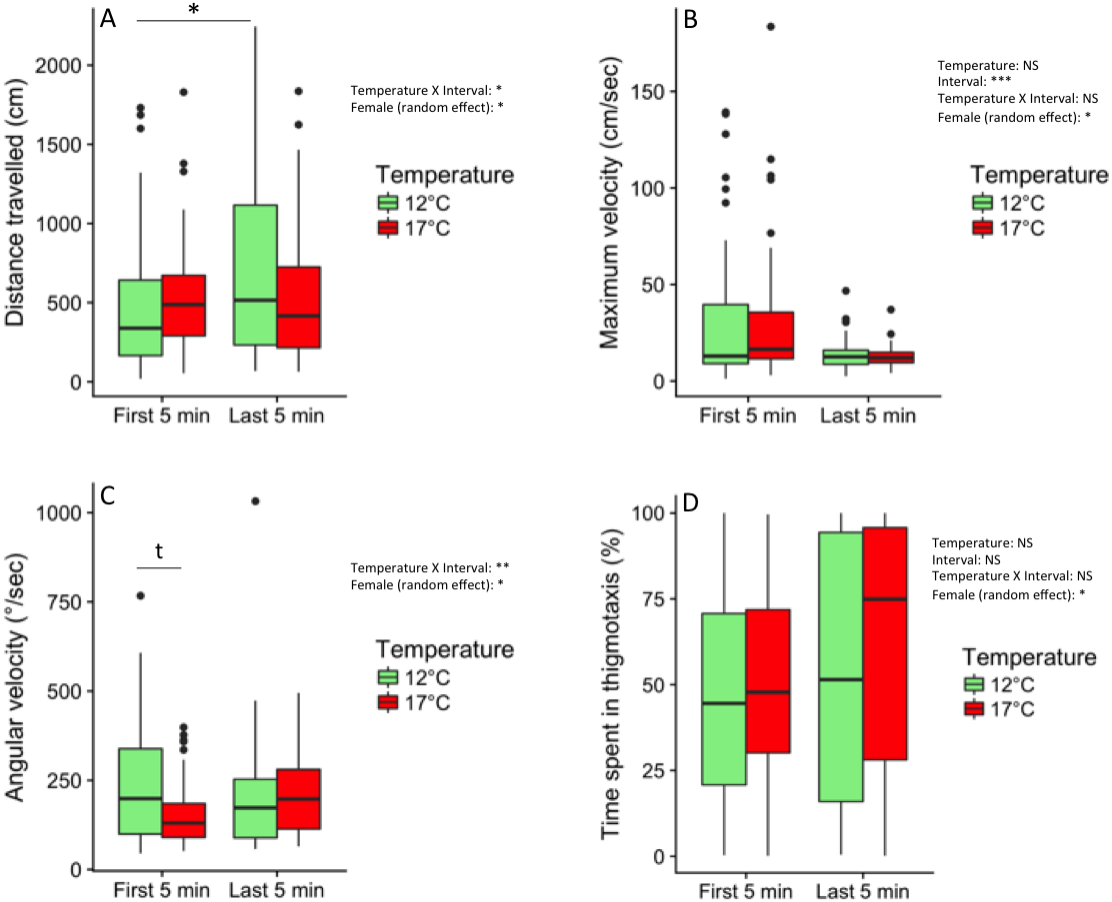
Swimming behaviour in novel-tank test. Swimming behaviour of 75–76 dpf progeny from mothers exposed to 12 °C and 17 °C before ovulation, video-filmed for 30 min in social isolation in a novel environment. Behaviours were recorded during the first 5-min interval of the test and the last 5-min interval of the test. (A) Total distance travelled (cm). (B) Maximum velocity (cm/sec). (C) Angular velocity (°/s). (D) Time spent in the border over the 5 min (% of time). Values are medians (quartiles: 25 and 75%) and data beyond the end of the whiskers are outliers and plotted as points. Significant main effects and interactions are indicated (NS: non significant, ^∗^*P* < 0.05, ^∗∗^*P* < 0.01, ^∗∗∗^*P* < 0.001). Random female effect is indicated (^∗^*P* < 0.05). Above the brackets, an asterisk indicates a significant difference (*P* < 0.05) and *t* represents a tendency (0.05 < *P* < 0.1), shown by post-hoc HSD-Tukey tests.

Offspring from thermally stressed mothers were less active than controls when individually introduced into a novel-tank (Fig. 3). When considering distance travelled, the temperature × interval interaction was significant (*F*_1,103_ = 5.26, *P* = 0.02, Fig. 3A). Post-hoc tests revealed a significant increase in the 12 °C group between the first 5 min and the last 5 min of the test (*P* < 0.05), that we did not observe in the 17  °C group. Variances associated with the random factors female and individual (4.56 and 4.34, respectively) were included in a confidence interval excluding 0, indicating that the random factors were significant. Maximum velocity did not differ between 12 °C and 17 °C (*F*_1,5_ = 0.08, *P* = 0.79, Fig. 3B). A significant global decrease was observed between the two intervals (*F*_1,201_ = 22.89, *P* < 0.001). No significant interaction was found (*P* = 0.56). The random factors female and individual were significant. When considering angular velocity, the temperature X interval interaction was significant (*F*_1,103_ = 7.60, *P* < 0.01, Fig. 3C). During the first 5 min, fish from 17 °C group tended to exhibit lower angular velocity than 12 °C fish (*P* = 0.06), as shown by post-hoc tests. No temperature or interval effects were found. No temperature effect, interval effect or significant interaction was found for time spent in thigmotaxis (Fig. 3D). The low variance (0.41) associated with the random factors female and individual indicated non-significant random effects. Models results are given in Table 1. Distance travelled and Angular velocity were negatively correlated (*r* =  − 0.57, *P* < 0.001).

### Offspring spatial learning

**Figure 4:**
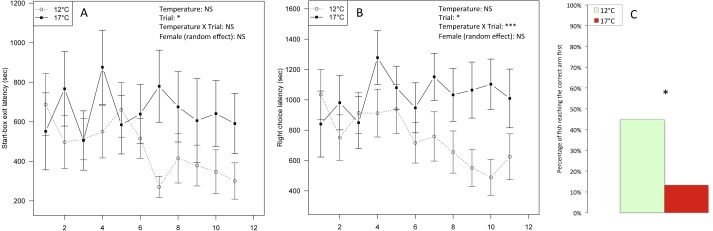
Spatial learning. (A) Latency (seconds) to leave the start-box and (B) latency to make the right choice by reaching the rewarded arm of a T-maze within 11 successive trials, lasting 1,800 s each, in progeny from mothers exposed to 12 °C and 17 °C before ovulation. Main effects and interactions are indicated (NS: non significant, ^∗^*P* < 0.05, ^∗∗∗^*P* < 0.001). Non-significant random female effect is indicated. Values are means and their associated mean standard error (SEM) (*n* = 5). (C) Percentage of fish choosing the rewarded arm first in less than 900 s in at least four out of the last seven trials in progeny from mothers exposed to 12 °C and 17 °C. ^∗^*P* < 0.05: significant difference between treatments (chi-square test).

Offspring from thermally stressed mothers were slower to locate the rewarded arm than controls, when tested in a T-maze (Fig. 4). We find a trial effect (*F*_1,343_ = 62.27, *P* < 0.05, Fig. 4A) when analysing the latency to leave the start-box, which decreased over time (i.e., cumulative number of trials). The temperature × trial interaction was not significant. The low variances (<0.001) associated with random factors female and individual indicated non-significant random effects. When analysing the latency to make the right choice, we found a significant temperature × trial interaction (*F*_1,347_ = 12.31, *P* < 0.001, Fig. 4B), indicating that 17  °C fish were slower to reach the rewarded arm following start-box exit. The random factors female and individual were not significant. Models results are given in Table 1.

The chi-square test showed a significant difference between the observed and the expected proportions of fish making the right choice in at least 4 out of the last 7 trials in 12 ° C (obs: 9/20, exp: 6.3/20) and in 17 °C (obs: 2/15, exp: 4.7/15) (*χ*^2^ = 4.05, *df* = 1, *P* < 0.05, Fig. 4C).

### Gene expression profiling in embryos with different maternal history

**Figure 5:**
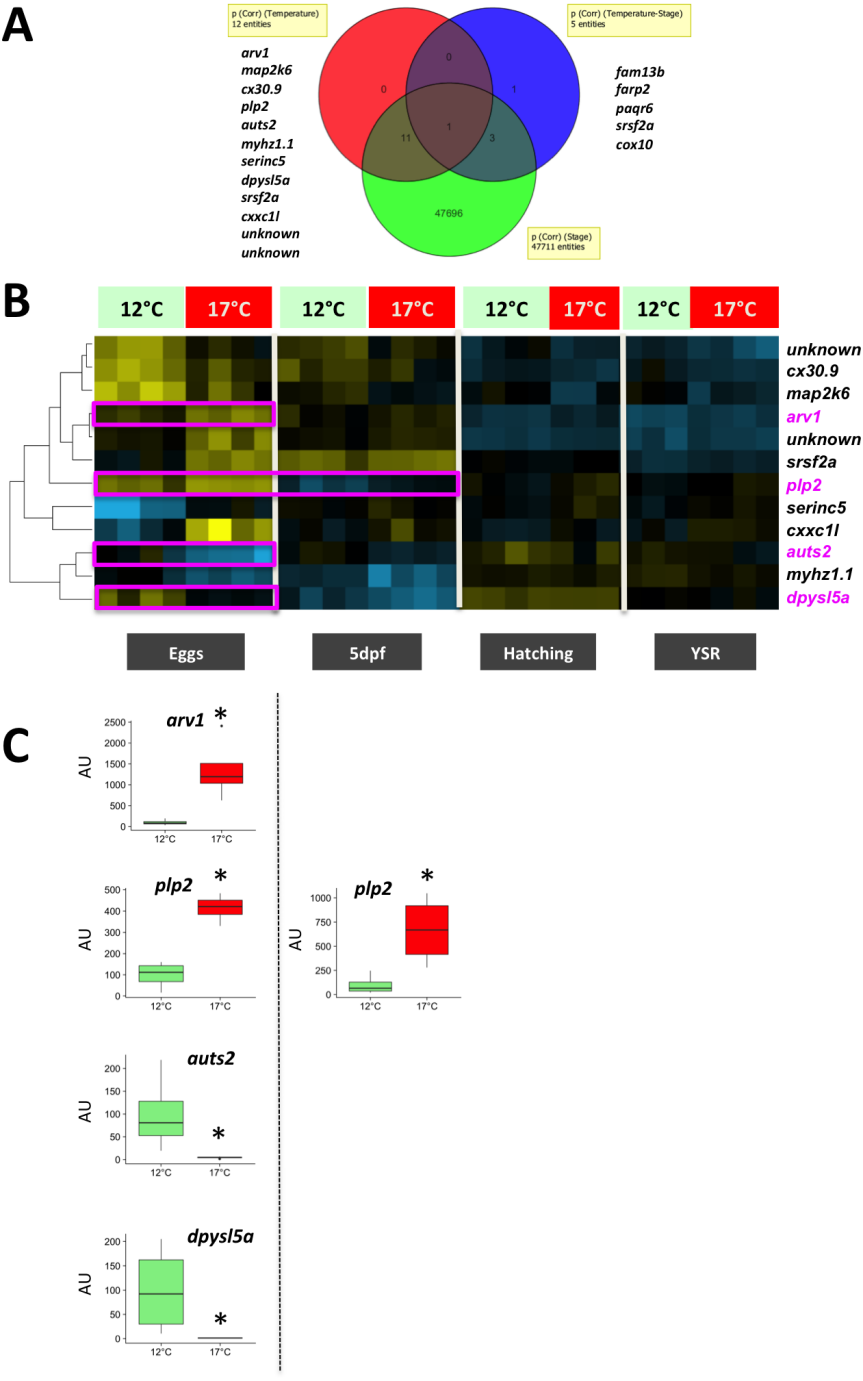
Microarray analysis of gene expression in eggs and progeny originating from mothers exposed to either 12 °C or 17 °C during the peri-ovulatory period. (A) Venn diagram representing the number of differentially expressed genes. Two-way-ANOVA performed using maternal temperature and developmental stage as fixed factors. Benjamini-Hochberg corrected *P*-values < 0.05. Gene symbols are shown when a significant effect was obtained for Temperature and Temperature × Stage interaction. All corresponding data are presented in Supplemental Information 1. (B) Supervised clustering analysis of the expression profiles of the 12 genes significantly dysregulated due to the temperature effect (A). Data were median-centered and an average linkage clustering was performed. Neurodevelopment genes and genes related to human cognitive disorders are shown in purple. (C). Boxplot representation of gene expression profiles of neurodevelopment genes (*auts2* and *dpyls5a*) and genes related to human cognitive disorders (*arv1* and *plp2*) corresponding to the data delineated in purple on (B). AU, arbitrary units.

Gene expression profiling was performed in eggs and throughout development after maternal exposure to either 12 °C or 17 °C. The ANOVA resulted in the identification of 47,711 differentially expressed genes throughout development. In contrast, a much lower number of genes were differentially expressed in response to maternal exposure to high temperature (Fig. 5A). Twelve genes exhibited a differential expression in response to temperature while only five genes were differentially expressed in response to temperature and among the developmental stages analysed (temperature × stage significant interaction: *P* <  0.05). A total of sixteen genes were thus significantly dysregulated during development in response to maternal exposure to high temperature, one gene (*srsf2a*) being present in both groups. Among these genes, several were of particular interest due to their role in neurodevelopment (*auts2, dpysl5*), neural disorder (*arv1*), and X-linked cognitive disability (*plp2*), as discussed below. Interestingly the expression profiling analysis (Fig. 5B) revealed that the differential expression between groups was especially marked in eggs, and to a lower extent at 5 dpf, while differences were more limited during further development (i.e., hatching and yolk-sac resorption stages). For *auts2* and *dpysl5* maternal mRNA abundance was dramatically lower when females were exposed to high temperature (*W* = 16, *P* < 0.05; Fig. 5C), while *arv1* exhibited an opposite pattern (*W* = 0, *P* < 0.05). Similarly, *plp2* abundance appeared higher in the 17 °C group in eggs and 5 dpf embryos (*W* = 0, *P* < 0.05; Fig. 5C). In order to rule out the possibility that the results could be due to the female exhibiting the most extreme phenotype (i.e., 98% mortality), statistical analyses were repeated after removing successively each individual female from the 17 °C group. We observed that the levels of significance (i.e., *p*-value) remained similar regardless of the female removed from the analysis.

## Discussion

Our aim was to investigate the effect of a thermal stress, applied to female rainbow trout during the peri-ovulatory period, on offspring behavioural phenotypes. As expected, the thermal stress triggered an increase in embryonic mortality, but not in the occurrence of malformed fry. In addition, fear-related locomotor responses to a novel environment were inhibited in 17 °C offspring, which might indicate impaired emotional responses. The thermal maternal stress also impaired spatial learning abilities in progeny. In consistency with these impaired behaviours, we observed a dysregulated expression of embryonic genes involved in neural and cognitive development revealed by a large-scale transcriptomic analysis.

### Maternal effects on embryonic survival and development

Our results are in full agreement with previous reports on the deleterious effect of high temperature exposure in peri-ovulatory period on offspring survival in salmonids (rainbow trout: (Aegerter & Jalabert, 2004), Atlantic salmon, *Salmo salar*: (King & Pankhurst, 2004; King et al., 2003; Taranger & Hansen, 1993), Arctic charr, *Salvelinus alpinus*: (Atse, Audet & De la Noüe, 2002). Despite small sample size (e.g., four females per treatment), differences between treatments were significant at hatching and yolk-sac resorption, but not at eyeing, which is also consistent with the results obtained by Aegerter & Jalabert (2004). In addition, body weight measured at 75 dpf tended to be lower in offspring originating from high temperature-exposed females. This is consistent with previous studies performed on fish, which showed lower offspring survival rates and impaired growth after maternal cortisol administration (Eriksen et al., 2007; Eriksen et al., 2015) or maternal stress exposure (Campbell, Pottinger & Sumpter, 1994; McCormick, 2009).

### Maternal effects on emotional responses

The novel-tank test consisted in observing immediate behavioural responses when fish were individually transferred into a novel environment, which is a context known to elicit acute stress responses in various vertebrates including salmonid fish species (Colson et al., 2018; Colson et al., 2015a; Kittilsen et al., 2009; Overli, Winberg & Pottinger, 2005; Rouger et al., 1998; Winberg et al., 2007). Our results show that fish originating from thermally stressed females were less reactive to the challenging situation than controls. Angular velocity, which represents erratic swimming and is commonly considered as an expression of fish anxiety (Blaser, Chadwick & McGinnis, 2010; Egan et al., 2009), tended to be lower in 17 °C fish during the first 5 min of the test. The maximum velocity, which is the first escape response commonly observed in isolated fish subjected to the novel-tank test (Champagne et al., 2010; Colson et al., 2015a), was dramatically increased in both groups immediately after the introduction into the novel tank (first 5 min). This observed ceiling effect is likely due to the strength of induced fear, which probably ruled out the possibility of detecting any difference between the two groups for this parameter.

While mean distance travelled increased at the end of the test for control fish suggesting a return to normal swimming pattern in this group, 17 °C fish exhibited a constant low swimming activity from the start to the end of the test. These findings suggest an impaired emotional reactivity, which is defined as the propensity to express fear when alarming stimuli occur (Désiré, Boissy & Veissier, 2002; Erhard et al., 2004; Favreau-Peigné et al., 2016). They are also consistent with the majority of studies performed in mammals, which showed reduced activity in the offspring of females subjected to different stressors during pregnancy (Fride et al., 1986; Fujioka et al., 2001; Masterpasqua, Chapman & Lore, 1976; Patin et al., 2004); (Suchecki & Palermo Neto, 1991), even though the stress was applied before fertilization in the present work. Interestingly, similar results were also found in fish (Eriksen et al., 2011; Espmark et al., 2008; Sopinka et al., 2014; Tierney, Patterson & Kennedy, 2009). For instance, Sockeye salmon (*Oncorhynchus nerka*) reared from mothers exposed to a chase stressor swam for shorter periods of time (Sopinka et al., 2014) and maternal cortisol exposure decreased time spent moving in offspring Atlantic salmon (Eriksen et al., 2011; Espmark et al., 2008).

In mammals, there is growing evidence that stress during pregnancy causes attention deficits and depressive disorders (Ronald, Pennell & Whitehouse, 2010; Talge, Neal & Glover, 2007), as well as impaired emotional behaviours of adult offspring (Fride et al., 1986; Shiota & Kayamura, 1989); Vallée et al., 1999; Zagron & Weinstock, 2006). The lack of behavioural reaction to the challenge observed in 17 °C fish resembles the depressive-like symptoms described in prenatally stressed rodents (Morley-Fletcher et al., 2003; Poltyrev et al., 2005). In these studies, animals do not further respond to stressful stimuli, and exhibit a decreased explorative behaviour and activity reflecting a form of resignation to an adverse uncontrollable situation. In a previous experiment, we noticed the absence of fear from a novel object (e.g., neophobia) in offspring from stressed females (Poisson et al., 2015). The absence of neophobia was likewise observed in suffering rainbow trout after being exposed to a nociceptive stimulus (Sneddon, Braithwaite & Gentle, 2003) and can be interpreted as a lack of attention for the environment. In the present study, the weaker emotional responses, as indicated by a decrease in angular velocity upon initial exposure to the novel tank and an absence of resumed ambulation at the end of the test might reflect attention alterations due to maternal stress.

Fish originating from thermally stressed mothers may be predicted to display a reduced ability to cope with their environment, since emotional alterations might be major disadvantages in adverse or changing environments (Bijlsma & Loeschcke, 2005). In rainbow trout, first feeding is a key-stage during which fear-related behaviour, such as fast-start swimming, ‘freezing’, hiding and exploring are essential traits for fry survival. Therefore, hypo-active behaviour, as shown in the present study, could have direct impacts on fish survival chances under natural conditions. It should also be mentioned that an absence of fear-related behaviour might be considered as a bold (or proactive) coping style (Andersson et al., 2013; Brown, Burgess & Braithwaite, 2007) that might have some adaptive functions in fluctuating and/or harsh conditions.

### Maternal effects on cognition

In the present experiment, we observed a learning deficit in 17  °C fish. Fry from mothers exposed to suboptimal temperature during late oogenesis were slower to locate the rewarded area in the spatial learning task and a very limited number of fish in this group (i.e., only two out of 15) reached the rewarded arm first during the last trials. This finding is consistent with studies performed in other oviparous species (birds: (Guibert et al., 2013; Lindqvist et al., 2007) and fish: (Eaton et al., 2015; Roche, McGhee & Bell, 2012), showing cognitive impairments in offspring of mothers stressed before fertilization compared to offspring of non-stressed animals. In three-spined sticklebacks, offspring of predator-exposed mothers located the food reward more slowly than offspring of unexposed mothers (Roche, McGhee & Bell, 2012). Female guppy (*Poecilia reticulate*) exposed to routine husbandry procedures that induced only a minimal elevation of cortisol, produced offspring that failed to associate a colour cue with food reward (Eaton et al., 2015). Conversely, in brook trout (*Salvelinus fontinalis*), maternal cortisol consumption and handling did not impact spatial learning or memory in 6 month-old offspring (Cortez Ghio et al., 2016). This inconsistency might indicate that maternal effects on fish cognition are context-dependent or different depending on the type of stress used. Except for the above examples, very few studies have investigated intergenerational effects on fish cognition, and to our knowledge our findings are the first to show that a thermal maternal stress is linked to emotional and cognitive impairments, even though the effects on emotional responses are weaker than the effects observed on cognition.

Several other possible causes exist that could explain the maternal effects on offspring traits observed here. One possible cause could be the differential offspring survival in control and 17 °C groups. In this case, experimental treatment would have selected fish exhibiting specific behaviours. It is also possible that the change in temperature, rather than the temperature itself, is responsible for the observed effects. Finally, it is possible that the difference between incubation conditions and the conditions that mothers might have anticipated when exposed to high temperature as suggested by Uller (2008) in an ecological context might also explain these results. Regardless of the explanation, our observations show that maternal exposure to high temperature ultimately results in offsprings exhibiting impaired emotional responses upon exposure to a novel environment and decreased learning performance.

In summary, fish originating from thermally stressed mothers were slower than controls in the spatial learning task and were fewer to choose the correct arm first. Cognitive abilities are critical for aquaculture fish since they need to anticipate specific events (e.g., food delivery) in order to reduce stress triggered by an unpredictable environment (Jones et al., 2012). Moreover, the ability to cope with repeated and fearful, but harmless, stimuli (e.g., repeated fishing linked to aquaculture practices) (Lieberman, 2000) can be extremely useful for fish in order to avoid chronic stress under aquaculture conditions. It is thus highly beneficial for cultured fish to enhance or at least to preserve learning abilities (e.g., conditioning and habituation). Cognitive processes remain a key component of fish welfare under breeding condition and this study reveals detrimental effects of maternal exposure to high temperature on these capacities in the offspring.

### Maternal effects on embryonic gene expression

Our results on the impact of thermal stress during the peri-ovulatory period (i.e., before fertilization) on offspring behaviour are similar to results obtained in mammals during pregnancy (Szuran, Zimmermann & Welzl, 1994; Talge, Neal & Glover, 2007; Vallée et al., 1999; Weinstock, 2005; Zagron & Weinstock, 2006). In humans, studies have shown that if a mother is stressed while pregnant, her child is at increased risk of having a range of problems, including emotional problems, attention deficits, and impaired cognitive development. These behavioural patterns resemble those observed in the present experiment. There is growing evidence for non-genetic effects of maternal experience on offspring gene expression in rodents (Weiss et al., 2011), and more recently in fish (Mommer & Bell, 2014). Here, we used a robust methodology (i.e., microarray) and a conservative statistical approach to reveal the most relevant molecular players despite the low number of females that simultaneously ovulated in both experimental groups. It is however noteworthy that the number of differentially expressed genes was much lower than what is usually observed in egg/embryo transcriptome analyses (Bonnet, Fostier & Bobe, 2007b; Fernandez-Diez et al., 2015; Mommens et al., 2014; Mommer & Bell, 2014; Zarski et al., 2017). It is also possible that only a limited number of genes are dysregulated in response to high temperature during the preovulatory period and/or that only a few genes are associated with the differences in behavioral phenotypes observed here. This also suggests that these genes play a key role in the behavioural differences observed between the two experimental groups. Indeed, among the sixteen differentially expressed genes, four genes are known to participate in neurodevelopment (*auts2, dpysl5*) or associated with neural/cerebral disorders (*arv1*) and × linked cognitive disability (*plp2*). In humans, *AUTS2* is officially named *activator of transcription and developmental regulator* according to the official gene nomenclature (HGNC:14262 https://www.genenames.org/). It was previously named *autism susceptibility candidate 2* (Sultana et al., 2002). The human *AUTS2* locus is associated with a wide diversity of neurological disorders, indicating that AUTS2 is involved in neurodevelopment (see Hori & Hoshino, 2017 for review). Several forms (splice variants) of the genes are expressed in the mouse during development, including during *in utero* development (Gao et al., 2014; Hori et al., 2014). In zebrafish, *auts2* is also embryonically expressed and found in the forebrain, midbrain and hindbrain at 24 h post-fertilization (Oksenberg et al., 2013). This early embryonic pattern in zebrafish and mouse is consistent with the embryonic expression profile reported here throughout rainbow trout development. Interestingly, *Auts2* expression in the mouse brain is especially high in regions associated with higher cognitive functions, including in the prenatal brain (Bedogni et al., 2010). Functional analyses conducted in zebrafish (*Danio rerio*) confirmed the major role played by *auts2* in fish neurodevelopment (Oksenberg et al., 2013). Knock down of *auts2* in zebrafish resulted in considerably less developing neurons in the optic tectum, retina, and cerebellum. Interestingly, observed phenotypes were less severe when the morpholino (MO) used was directed against a splice junction rather than the translation initiation site, indicating that maternally-inherited *auts2* mRNA played an important role in Auts2-mediated neurodevelopment. Together, these observations are fully consistent with our data, especially the profiles of *auts2* maternal RNA shown in Fig. 5B and suggest that the intergenerational effects of maternal exposure to high temperature could be mediated, at least in part, by differences in egg content in *auts2* messenger RNA. Data in mouse and zebrafish indicate that Auts2 acts as a transcriptional regulator for neural development through interactions with several genes related to brain development and neurological disorders. More specifically, Auts2 appears to be participating in neuronal migration and neurite extension and is critical for the acquisition of neurocognitive function (see Hori & Hoshino, 2017 for review). Behavioural phenotypes observed in Auts2 heterozygous mutant mice are characterized by lower anxiety-like behaviour and impaired memory (Gao et al., 2014; Hori et al., 2015). These phenotypes are similar to the phenotypes observed here after maternal exposure to high temperature and characterized by weaker emotional responses (i.e., lower angular velocity and absence of locomotor activity modifications under stressful situation) and impaired learning abilities (i.e., slower to locate a food-reward than controls in a T-maze). In addition to *auts2*, we also observed the dysregulation of *dpysl5, (dihydropyrimidinase-like 5*) a member of CRMP (collapsing response mediator protein) family thought to be involved in neural development (Veyrac et al., 2011). Together these observations strongly suggest that the dysregulation of neurodevelopment genes expression, especially *auts2,* but also *dpysl5*, in eggs and embryos participate in mediating the intergenerational effects on offspring behaviour observed here after exposing rainbow trout females to high temperature.

The transcriptomic analysis also revealed the differential expression, in response to maternal exposure to high temperature, of genes associated with neural/cerebral disorders (*arv1*) and X-linked cognitive disability (*plp2*). In an attempt to better understand genes affecting human brain function, a recent whole-exome sequencing study in 143 families resulted in the identification of 68 recessive genes associated with neurological disorders (Alazami et al., 2015). Among those genes was *ARV1*, which was also associated with autosomal recessive epileptic encelopathy in another study (Palmer et al., 2016). We also observed a dysregulation of *plp2* in response to maternal exposure to high temperature. In humans a polymorphism in *PLP2* (*Proteolipid protein 2*) promoter was associated with X-linked Mental Retardation (XLMR) (Zhang et al., 2007). While the roles of *arv1* and *plp2* in fish are currently unknown, the identity and suspected roles of these genes in humans are consistent with the differential abundance of the gene and the weaker emotional responses and impaired learning abilities observed in the present study.

The genome-wide transciptome analysis also revealed the dysregulation of several other genes, including a so far uncharacterized gene (*cxxcl1l*) that exhibits a strong differential expression in eggs from different maternal origin. These genes are likely to mediate, or at least to participate, in the intergenerational effect of maternal exposure to high temperature observed here. Further analyses are needed to decipher the specific contribution of these genes to the phenotypes reported here.

## Conclusions

Together, our results revealed the dysregulation of several genes that are important for the development of cognitive abilities in response to maternal exposure to high temperature. This is especially true for *auts2*, a key gene for neurodevelopment, and critical for the acquisition of neurocognitive function in fish and mammals. In addition to *auts2*, our analysis revealed the dysregulation of another neurodevelopment gene (*dpysl5)* as well as genes associated with cognitive disorders in humans (*arv1*, *plp2*). Our study also revealed that some of the observed intergenerational effects are associated with a major dysregulation of several maternally-inherited mRNAs accumulated into the egg. The identity of these genes is consistent with the behavioural phenotypes observed in fry originating from thermally stressed mothers. Fry exhibited impaired emotional responses and reduced learning performance, which would be a major disadvantage for wild or cultured fish under suboptimal or fluctuating environments. Additional studies aiming at characterizing possible epigenetic modifications, gene expression and neurotransmitters activity in target brain structures are still needed to further understand the mechanisms mediating the observed intergenerational effects.

##  Supplemental Information

10.7717/peerj.6338/supp-1 Supplemental Information 1 Mortality data

10.7717/peerj.6338/supp-2 Supplemental Information 2 Malformed embryos data

10.7717/peerj.6338/supp-3 Supplemental Information 3 Novel-tank test data

10.7717/peerj.6338/supp-4 Supplemental Information 4 Learning data

10.7717/peerj.6338/supp-5 Figure S1 Types of malformations Effects of rearing temperature before ovulation (12° C and 17° C) on the occurrence of different types of embryonic malformation (%) at yolk-sac resorption. T, torsion; YSD, Yolk-sac resorption defects, O, Others.

## Additional Information and Declarations

### Microarray Data Deposition

The following information was supplied regarding the deposition of microarray data: Data is available in the Gene Expression Omnibus (GEO) database under accession number GSE113377.

### Data Availability

The following information was supplied regarding data availability: The raw data are provided in a Supplemental File.
