# Supplementary figures and images for: Maternal temperature exposure impairs emotional and cognitive responses and triggers dysregulation of neurodevelopment genes in fish
Source: PeerJ. 2019 Jan 31;7:e6338. doi: 10.7717/peerj.6338 (PMC6360074; doi:10.7717/peerj.6338)

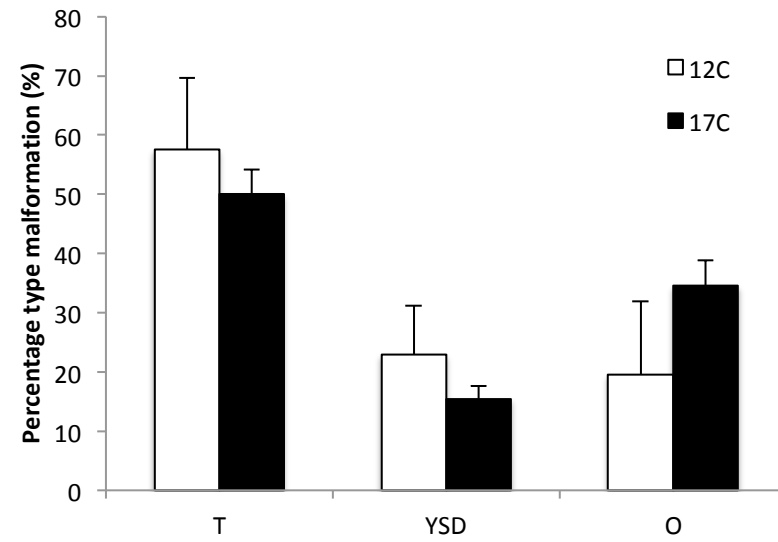

Supplement: Figure S1 — Effects of rearing temperature before ovulation (12° C and 17° C) on the occurrence of different types of embryonic malformation (%) at yolk-sac resorption. T, torsion; YSD, Yolk-sac resorption defects, O, Others. [file peerj-07-6338-s005.pdf]
